# Saved by anticoagulants: Incidental discovery of a misplaced defibrillator lead 6 years after implantation. Inadvertent lead placement inside the left ventricular cavity

**DOI:** 10.1002/ccr3.4062

**Published:** 2021-03-20

**Authors:** Abdelrahman Osman, Ali Ahmad

**Affiliations:** ^1^ College of Arts and Sciences The Ohio State University Columbus OH USA; ^2^ Department of Cardiovascular Imaging Mercy Tiffin Cardiovascular Center Tiffin OH USA

**Keywords:** cardiovascular disorders

## Abstract

Device insertion is a common cardiovascular procedure. Devices are implanted into the right heart, acting as a safeguard against systemic thromboembolism. Lead insertion into the left ventricle is rare, but carries dangerous consequences of thromboembolic events. Diagnosis and intervention of an inadvertently placed lead is essential. This is a case of a defibrillator lead inadvertently inserted into the left cavity, discovered 6 years after implantation.

## Graphical abstract

Pacemakers are typically used to restore normal heart rhythm to the heart. While typically found screwed in the right ventricle, pacemaker leads may sometimes travel through the walls of the heart and become lodged in the right ventricle. This complication must be resolved immediately due to the high risk of thromboembolism.

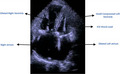

## 1 INTRODUCTION

**FIGURE 1:**
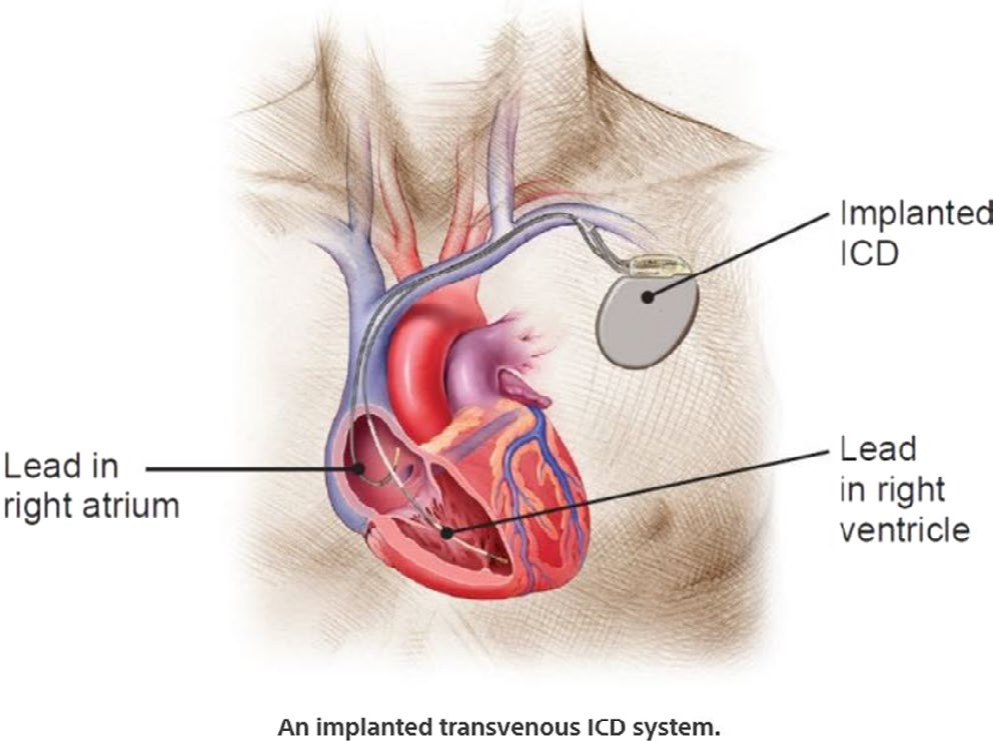
Normal ICD placement, in which the lead is screwed into the right ventricle

Placement of a pacemaker or an implantable cardioverter‐defibrillator (ICD) is a minimally invasive procedure. With access through the left subclavian, cephalic, axillary, or femoral vein, the latter usually in the setting of a temporary need for device therapy, the ICD leads are placed into the heart and screwed into position in the right ventricle (Figure 1).^1^ Inadvertent placement of a pacemaker/defibrillator lead, albeit rare, has been observed in different situations, but a device lead inside the left ventricular cavity carries a special risk of a thromboembolic event. Hence, prompt identification and early management of misplaced leads inside the left ventricular cavity are essential. The frequency of this complication is unknown, but we believe it is markedly underreported. The most common cause of misplaced right ventricular lead into the left ventricular cavity is lead migration from the right ventricle through the interventricular septum (IVS). Occasionally, an epicardial left ventricular lead inserted into a branch of the coronary sinus can perforate into the endocardium then into the left ventricular cavity in case of cardiac resynchronization therapy (CRT). Rarely, a pacemaker/defibrillator lead may travel via a congenital defect in the interatrial septum (IAS) to the left side of the heart.

Inadvertent pacemaker lead placement can be diagnosed using lateral and antero‐posterior (AP) chest X‐rays and further confirmed by a 12‐lead electrocardiogram (ECG) exhibiting a right bundle branch block (RBBB) pattern rather than the expected left bundle branch block (LBBB) appearance on ventricular pacing mode.^2^, ^3^ A 12‐lead ECG is not helpful if the patient has only atrial pacing or no pacing at baseline like in cases of sinus node dysfunction without atrioventricular node disease or in cases of ICD implantation for primary prevention of sudden cardiac death (SCD). Device interrogation at bedside may give a clue of inadvertent lead placement like high pacing threshold or even high impedance. Further imaging including echocardiogram, cardiac computerized tomography (Cardiac CT), or transesophageal echocardiogram (TEE) may be needed to establish the diagnosis and to identify the mechanism of lead migration as clinically indicated.

Diagnosis can occur any time after implantation and approaches differ depending on patient clinical status and time of displacement. In early displacement, revision with repositioning of the lead is possible because the lead has not yet been fixed in the heart. However, with late displacements, repositioning carries unknown and unnecessary risks.^4^ We are presenting a rare case of a dual‐chamber defibrillator placement where the shock lead was erroneously inserted into the left ventricular cavity via a congenital defect. Unfortunately, this was discovered 6 years after initial implantation, but luckily no thromboembolic complications happened because patient was on chronic anticoagulation throughout that time.

## 2 CASE PRESENTATION

**FIGURE 2:**
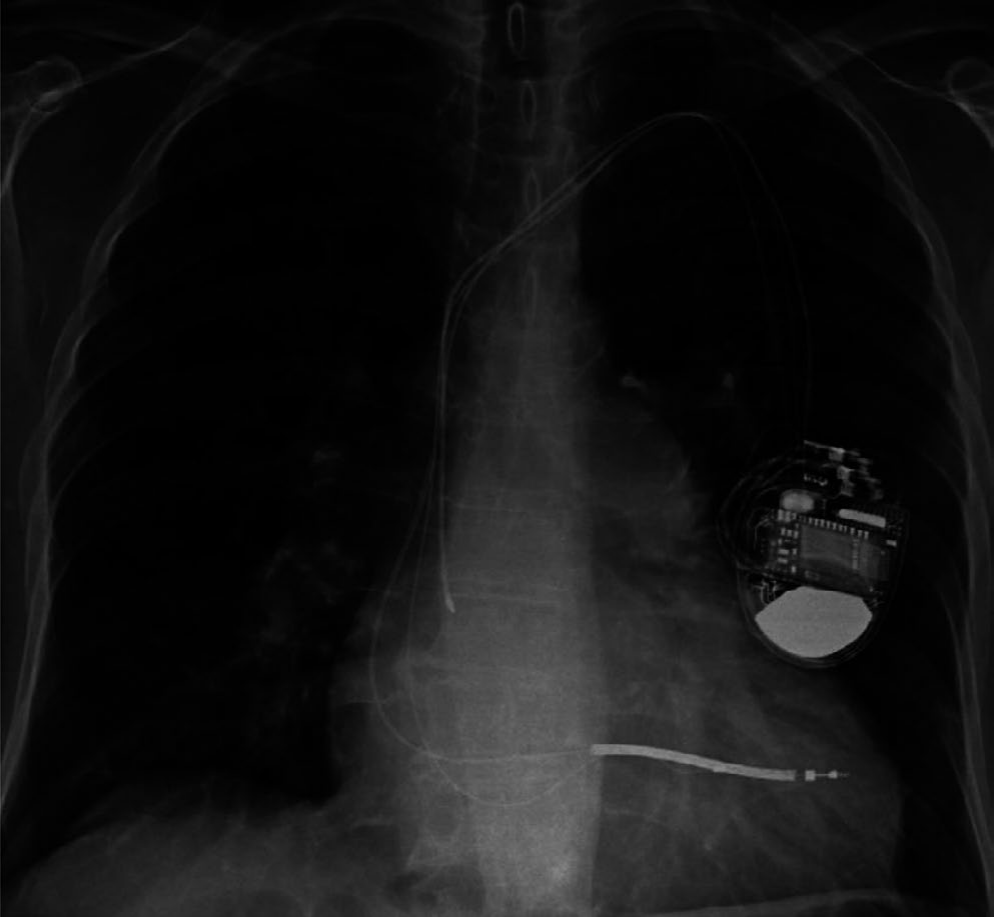
AP chest X‐ray showing ICD shock lead in an apparently good position

A 76‐year‐old man with history of ischemic cardiomyopathy and paroxysmal atrial fibrillation who underwent a Dual‐Chamber Medtronic Implantable Cardioverter‐Defibrillator (ICD) placement 6 years earlier in an outside facility presented to the emergency room with worsening shortness of breath. Initial workup in the emergency room revealed acute on chronic combined congestive heart failure. An AP chest X‐ray film was interpreted by the radiologist as cardiomegaly with pulmonary vascular congestion and satisfactory ICD lead position (Figure 2). No lateral view was obtained at the time.

**FIGURE 3:**
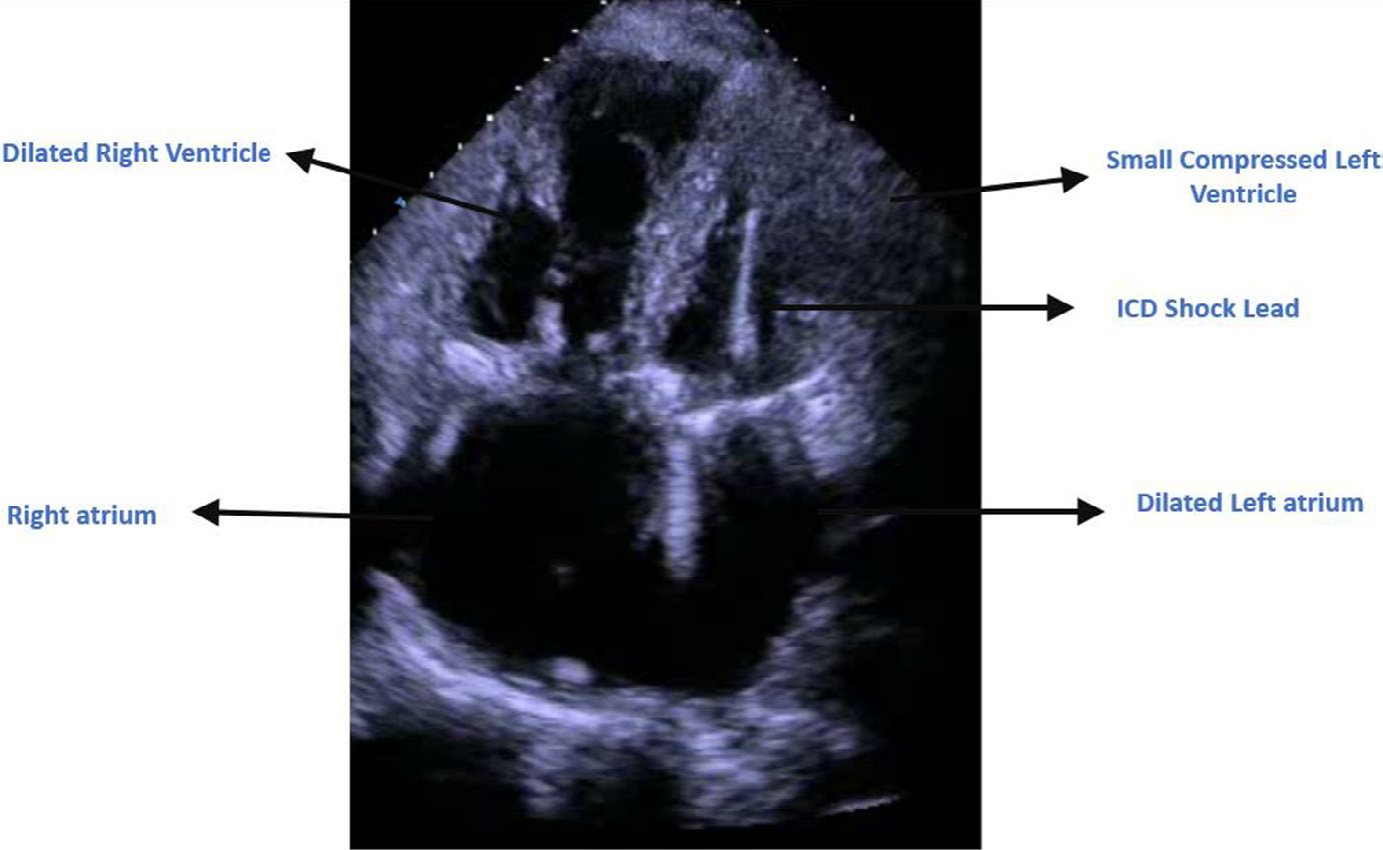
Apical 4‐chamber view showing the ICD shock lead going through the mitral valve into the left ventricular cavity. Note the moderately to severely dilated right ventricle with hypertrophied wall indicating significant pulmonary hypertension. Bi‐atrial enlargement is also noted

**FIGURE 4:**
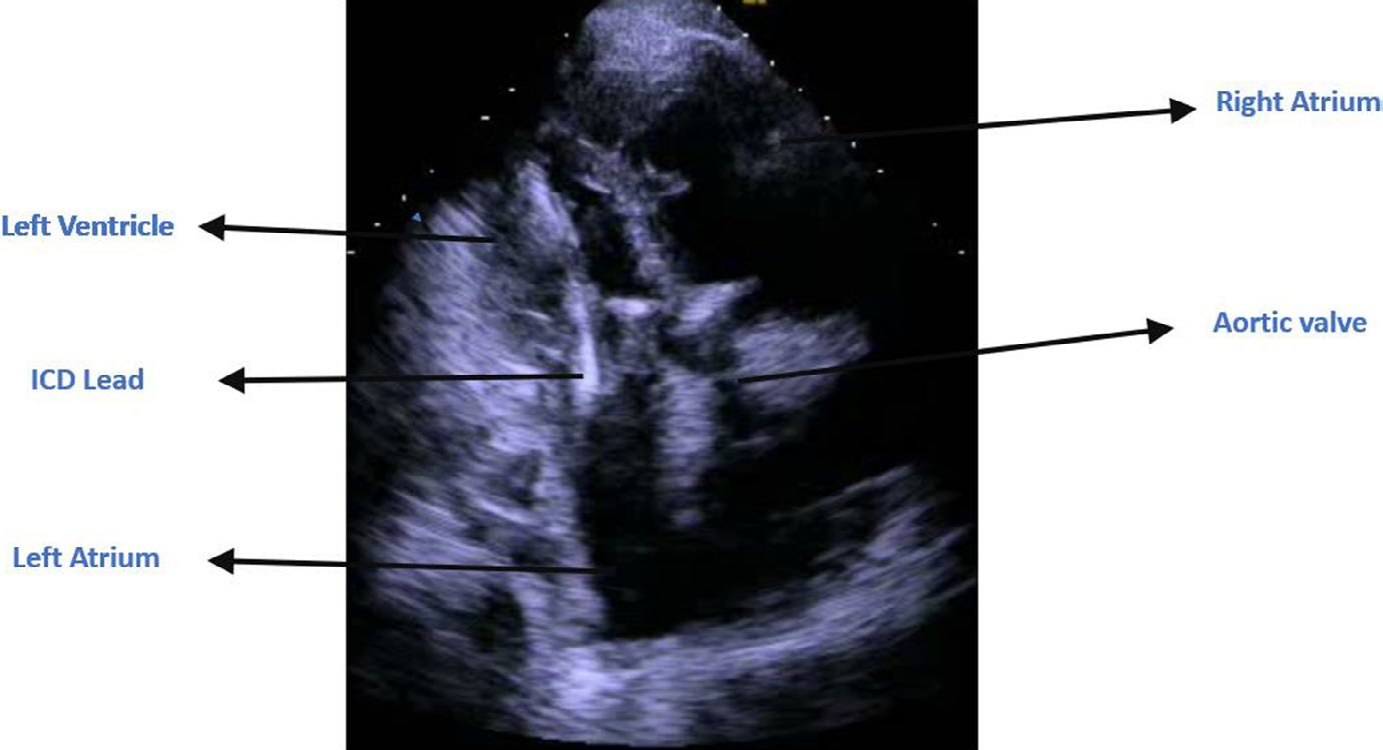
Apical 3‐chamber view showing the ICD shock lead clearly going through the mitral valve into the left ventricle

**FIGURE 5:**
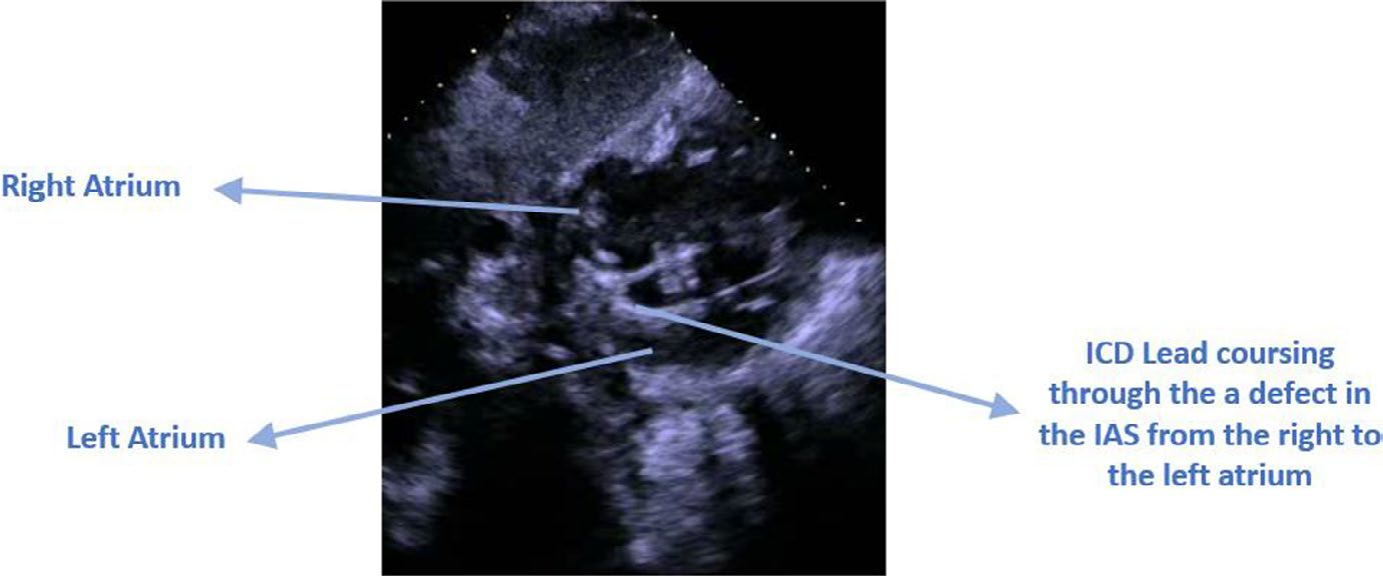
Subcostal view showing the ICD shock lead coursing through a sinus venosus defect from the right atrium (RA) to the left atrium (LA) into the mitral valve (MV)

**FIGURE 6:**
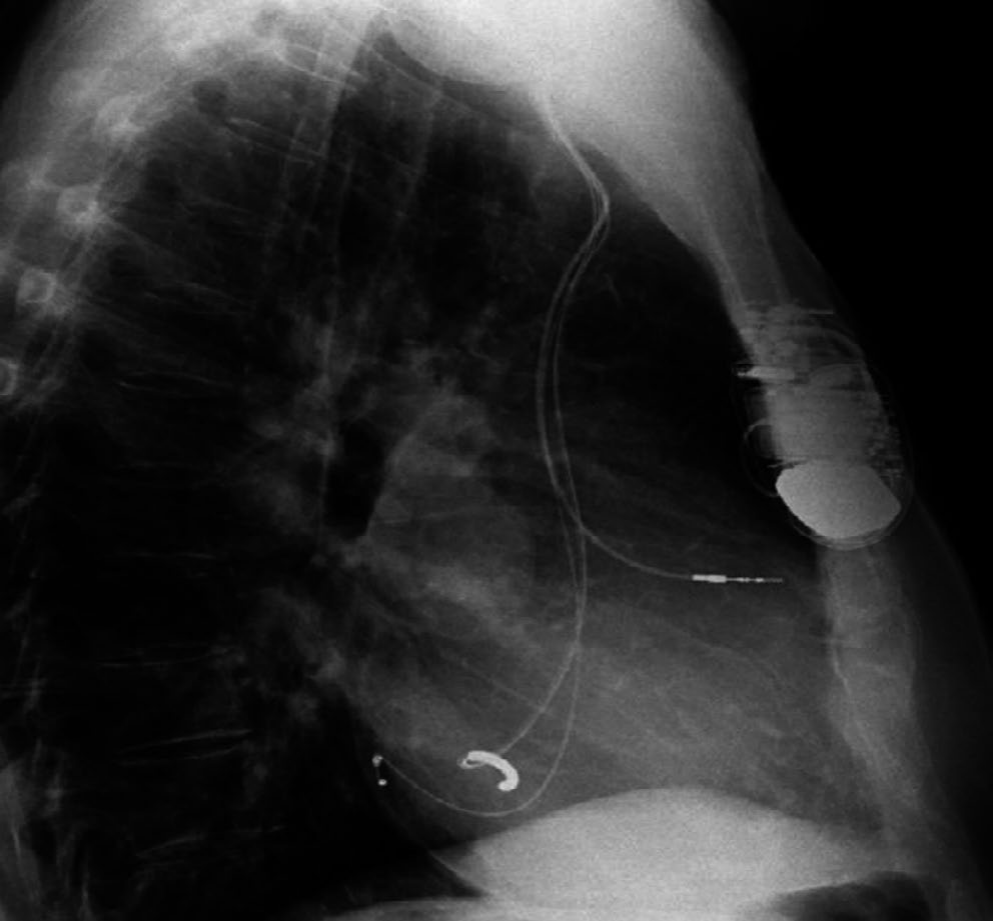
Lateral chest X‐ray of a patient with inadvertent left ventricular lead placement. The lead is clearly seen posteriorly, indicating migration to the left ventricle

Bedside Echo showed severely reduced left ventricular systolic function with dilated right ventricle and moderate‐to‐severe pulmonary hypertension. Surprisingly, the ICD lead was clearly going from the left atrium through the mitral valve to the left ventricular cavity (Figures 3, 4, and 5). A lateral chest X‐ray view revealed that the ICD shock lead tip is directed posteriorly, indicating its presence in the left ventricular cavity (Figure 6). A transesophageal echocardiogram showed the ICD lead went from the superior vena cava to the right atrium, crossing through a small sinus venous atrial septal defect to the left atrium, and into the left ventricular cavity via the mitral valve. Luckily, the patient was on chronic anticoagulant because of chronic atrial fibrillation, so no thromboembolic complications were reported. Interestingly enough, the patient had two prior trans‐thoracic echocardiograms that were interpreted by the same cardiologist without mention of abnormal ICD lead placement. Furthermore, prior ICD interrogation reports from his primary cardiologist showed chronically elevated shock lead impedance but pacing threshold and sensing were good. No therapy was needed since device implantation.

Because extraction of a chronically implanted ICD lead carries a high risk of major cardiovascular complications (2%‐4%) including vascular injury, cardiac perforation resulting in cardiac tamponade, and occasionally thromboembolic complications or even death, and because the patient was chronically anticoagulated, the decision is made to continue conservative management.

## 3 DISCUSSION

Implantation of a pacemaker or defibrillator is a common procedure to treat heart rate and rhythm abnormalities. Single lead ICDs are used to prevent sudden cardiac death in patients at high risk of life‐threatening cardiac arrhythmia by sensing and then delivering electric shock to restore normal cardiac rhythm. They are typically placed in the right ventricular cavity. However, 27% of all complications from ICD insertions are due to lead dislodgment or unsatisfactory position.^4^ The migration of a lead occurs in different ways, but passage is most commonly through an atrial septal defect (ASD) or patent foramen ovale. Although reported in various cases, inadvertent lead placement inside the left ventricle is uncommon, but can lead to dangerous thromboembolic (TE) events.^5^ TE events are the result of thrombus formation around the implantation site and can occur from days to years after implantation.^4^ Other complications of inadvertent placement of an ICD lead in the left ventricle are pericardial effusion, endocarditis, vascular damage, and peripheral arterial thrombosis.^6^

Diagnosis of a misplaced lead in the left ventricle requires high index of suspicion and immediate action. A misplaced lead in the left ventricle creates an RBBB‐pattern on an ECG and is often the most important tool in diagnosis.^2^, ^3^ Due to the similarity of this pattern with right ventricle dilatation and coronary sinus pacing, a confirmatory test is typically used, with AP and lateral chest X‐rays being the primary instrument. While a correctly placed lead is seen with a slight bowing at the right ventricle on an AP view, a misplaced lead is typically seen to the left and farther superior. On a lateral projection, a correctly placed ICD lead tip is located anteriorly, but a misplaced lead's tip points posteriorly.^3^ As in our case, a transesophageal echocardiogram can show a pacemaker lead crossing from the right atrium to the left atrium, then through the mitral valve before settling in the left ventricle.

Management of an inadvertent left ventricle lead depends heavily on time after implantation. Early detection of a misplaced lead allows for lead extraction, reducing the risk for TE events and avoiding the need for lifelong anticoagulation.^7^ If diagnosis is delayed, however, the lead becomes fixed in the heart and anticoagulation is needed to avoid TE events. If the patient is young and healthy, extraction can also be considered, but can lead to significant vascular complications.

## 4 CONCLUSION

Inadvertent pacemaker or defibrillator lead placement can lead to serious and occasionally life‐threatening complications and can be overlooked for a long period of time. Very careful interpretation of the ECG and device data “Pacing threshold, sensing and impedance” in addition to appropriate use of the available imaging modalities can lead to early diagnosis and treatment and can save the patient from such serious complications.

## CONFLICT OF INTEREST

The authors declare no conflicts of interest.

## AUTHOR CONTRIBUTIONS

AA: analyzed and studied patient complaints, images, and eventual diagnosis, as well as reviewed all drafts of the article. AO: wrote main manuscript and critically reviewed all drafts.

## ETHICAL APPROVAL

Written with consent of the patient.

## Data Availability

Data sharing is not applicable as no new datasets were generated or analyzed.
